# Identification of *RELN* variation p.Thr3192Ser in a Chinese family with schizophrenia

**DOI:** 10.1038/srep24327

**Published:** 2016-04-13

**Authors:** Zhifan Zhou, Zhengmao Hu, Lu Zhang, Zhaoting Hu, Haihong Liu, Zhening Liu, Juan Du, Jingping Zhao, Lin Zhou, Kun Xia, Bengsha Tang, Lu Shen

**Affiliations:** 1 Department of Neurology, Xiangya Hospital, Central South University, Changsha 410008, China; 2 Hunan Province Key Laboratory in Neurodegenerative Disorders, Central South University, Changsha 410008, China; 3 State Key Laboratory of Medical Genetics, Changsha 410008, China; 4 BGI-Shenzhen, Shenzhen, Guangdong Province, 518083, China; 5 Department of Computer Science, City University of Hong Kong, Hong Kong 999077, China; 6 Mental Health Centre, Xiangya Hospital, Central South University, Changsha 410008, China; 7 Mental Health Institute, Second Xiangya Hospital, Central South University, Changsha 410011, China

## Abstract

Schizophrenia (SCZ) is a serious psychiatric disease with strong heritability. Its complexity is reflected by extensive genetic heterogeneity and much of the genetic liability remains unaccounted for. We applied a combined strategy involving detection of copy number variants (CNVs), whole-genome mapping, and exome sequencing to identify the genetic basis of autosomal-dominant SCZ in a Chinese family. To rule out pathogenic CNVs, we first performed Illumina single nucleotide polymorphism (SNP) array analysis on samples from two patients and one psychiatrically healthy family member, but no pathogenic CNVs were detected. In order to further narrow down the susceptible region, we conducted genome-wide linkage analysis and mapped the disease locus to chromosome 7q21.13-22.3, with a maximum multipoint logarithm of odds score of 2.144. Whole-exome sequencing was then carried out with samples from three affected individuals and one unaffected individual in the family. A missense variation c.9575 C > G (p.Thr3192Ser) was identified in *RELN*, which is known as a risk gene for SCZ, located on chromosome 7q22, in the pedigree. This rare variant, as a highly penetrant risk variant, co-segregated with the phenotype. Our results provide genetic evidence that *RELN* may be one of pathogenic gene in SCZ.

Schizophrenia (SCZ [OMIM 181500]) is a severe psychiatric disease that begins in adolescence or early adulthood, characterized by positive symptoms (e.g. delusions, hallucinations, and disorganized thinking), negative symptoms (e.g. flat affect, loss of spontaneity, diminished initiative and capacity for pleasure, and impaired volition), numerous cognitive dysfunctions of varying severity, and mood disturbances. The lifetime prevalence of SCZ is approximately 1% in the general population worldwide. Genetic causes play an important role in the pathogenesis of SCZ, which is strongly suggested by high heritability estimates (0.64 in a national family study^1^, 0.81 in a meta-analysis of twin studies^2^).

Pedigrees with multiple affected individuals serve as a valuable tool for discovery of rare and highly penetrant mutations which upon identification will shed light onto biological pathways involved in the pathogenesis of diseases. In pedigree studies, linkage analysis as traditional positional cloning method has been used to identify causal genes of several rare monogenic diseases. This method is also applicable to complex neuropsychiatric diseases, etc, Alzheimer’s disease^3^. Based on linkage analysis, St Clair *et al.*^4^ discovered a balanced translocation (1;11)(q42.1;q14.3) segregated with SCZ and related psychiatric disorders in a large and unique Scottish family^4^. As translocation directly disrupts the disrupted-in-schizophrenia 1 gene (DISC1), it is usually considered one of the candidate genes for susceptibility to psychiatric illnesses^5^. Apart from the 1q42 region, other suspected regions, including 1q21-22, 5q21-q33, 6p24-22, 6q21-25, 8p21-22, 10p15-p11, 13q32-34, and 22q11-12, have been reported to correlate with SCZ using linkage analysis^6^. With the application of high-throughput sequencing, a few studies have employed complementary approaches (e.g. linkage analysis, exome sequencing in pedigrees with multiple affected individuals) to identify private, highly penetrant mutations that co-segregate with psychiatric disease^7^,^8^,^9^. A deletion at the *SLC1A1* glutamate transporter gene has been reported to co-segregate with schizophrenia and bipolar schizoaffective disorder in an extended five-generation family^7^. Most specifically, exome sequencing of multiplex pedigrees further supported the N-methyl-D-aspartate (NMDA) receptor hypofunction hypothesis of SCZ^8^. These studies have generated interesting data on contributions of rare genetic variations to psychiatric disease risk.

Because of the need for larger sample sizes to achieve sufficient statistical power to derive any disease association, recent genetic studies of SCZ have shifted away from family-based to population-based studies. Genetic risk for SCZ is conferred by common variants and rare variants. Genome-wide association studies (GWAS) based largely on common variants have identified over 100 schizophrenia risk loci, some of which are the most reproducible and reliable ones in the field of SCZ genetics^10^,^11^ (PGC is now known as the Psychiatric Genomics Consortium). But contribution of common variants toward schizophrenia risk has been modest. Compared with SCZ-associated common variants of small effect, rare variants with relatively high penetrance significantly increase risk of illness^12^,^13^,^14^,^15^,^16^,^17^. Very large number of rare variants, including copy number variants (CNVs)^12^,^13^,^14^,^15^ and single-nucleotide variants (SNVs)^16^,^17^, were involved in the etiology of SCZ. For example, CNVs finding from an enlarged case-control sample indicated that the strong functional links, between the major inhibitory GABAergic and excitatory glutamatergic systems, may be proposed to explain some of disease mechanisms^18^. In an analysis of exome sequencing data of 2,536 SCZ cases and 2,543 controls, Purcell *et al.*^19^. estimated that rare (less than 1 in 10,000), disruptive mutations account for a broadly comparable proportion of SCZ risk as do CNVs^19^. Although recent researches have considerably advanced our understanding of the genetic architecture of SCZ, the complete spectrum of SCZ susceptibility genes has not been completely discovered yet.

In the present study, we enrolled a large Chinese family with multiple affected subjects with SCZ in which its genetic transmission appeared most consistent with autosomal-dominant inheritance. Thus, we used a combined strategy to determine the genetic basis of SCZ in this family, involving CNV genome-wide microarray analysis, linkage analysis, and exome sequencing.

## Results

### Clinical manifestation

**Figure 1:**
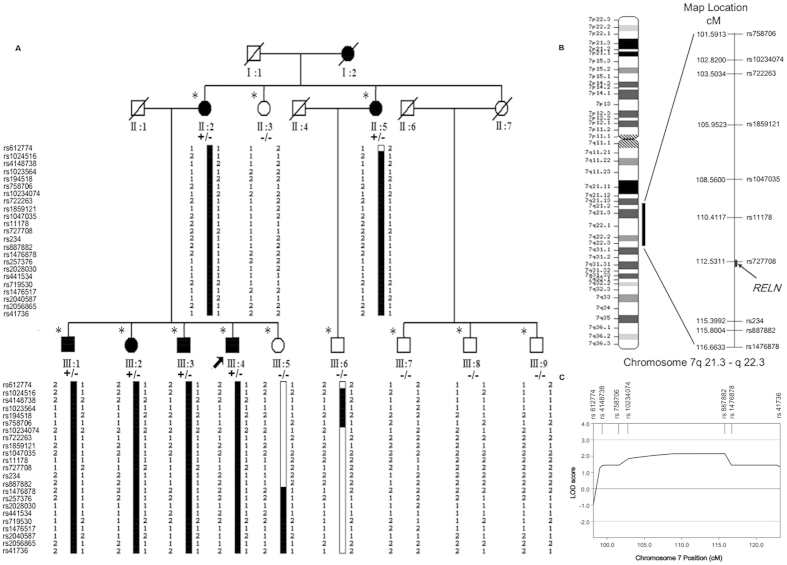
Linkage analysis mapped the schizophrenia (SCZ) family to a region on chromosome 7q21.13-22.3. (**A**) A 3-generation Chinese family with autosomal-dominant SCZ is shown and haplotype analysis is indicated on chromosome 7 using partial single nucleotide polymorphism (SNP). Squares and circles indicate males and females, respectively. The black symbols represent the affected members and open symbols represent the unaffected individuals. The diagonal line indicates a deceased family member and the black arrow indicates the proband. Minus indicates the wild-type allele; plus indicates the heterozygous missense variant in *RELN* (c.9575 C > G). The family members enrolled in this study are marked with asterisks. Names of SNPs are given on the left. Haplotype alleles appear on one side. The black bar indicates the haplotype assumed to carry the disease allele. The bars with black and white haplotypes indicate a recombination event. (**B**) The diagram of chromosome 7 indicates the relative position of SCZ locus. The minimal linkage interval of SCZ is 15.072 cM, between rs758706 and rs1476878, on chromosome 7q21.13-22.3. The RELN position is indicated with a black arrow. (**C**) Multipoint logarithm of odds (LOD) scores for chromosome 7q are shown (frequency of 0.01 and 80% penetrance). SNPs locations are indicated by ticks at the top.

The proband (III:4) was a 47-year-old male, diagnosed with SCZ at the age of 36 years (Fig. 1A). He was born after an uneventful, full-term pregnancy, with normal birth weight and did not exhibit motor or verbal delay. He exhibited extremely severe positive symptoms at 36 years of age, including formication, auditory hallucinations of threatening and commanding voices, persecutory delusions, self-talk, and impulsive behavior. Treatment with sulpiride resulted in a significant improvement of his psychiatric symptoms, while discontinuation of sulpiride led to aggravation of symptoms. On examination at our facility, he showed moderate psychotic symptoms (auditory hallucinations and persecutory delusions) at the age of 42 years. Findings from general physical and neurological examinations were normal. On cognitive testing, the patient’s Minimum Mental State Examination (MMSE) score was 27 and Montreal Cognitive Assessment (MoCA) score was 20. Laboratory examinations were unremarkable. Magnetic resonance imaging (MRI) of the brain did not show gross structural abnormalities or significant atrophy in the hippocampus and frontal temporal lobe. The clinical features of the 6 affected patients included in this study are summarized in Table 1. The age of onset ranged from 31 to 39 years (mean 35 ± 3.0 years).

### CNVs detection

We analyzed DNA from two subjects with SCZ and one 70-year-old psychiatrically healthy control using Human660W-Quad Chip. From the total of 1189 CNVs, only 46 CNVs were consistent with clinical phenotype. After comparison with the Database of Genomic Variants (DGV, http://projects.tcag.ca/variation/), all the 46 CNVs referred to common copy number polymorphisms. Furthermore, no previously reported pathogenic large rare CNVs were identified in the affected individuals (II:5 and III:1)^18^,^20^,^21^,^22^. Therefore, we concluded that rare CNVs were not responsible for schizophrenia in this family.

### Linkage mapping

Genome-wide linkage mapping with SNP markers covering all chromosomes was undertaken to localize the mutation responsible for the disease. Parallel inspection of SNP data from the family identified a single shared region on chromosome 7q21.13-q22.3, flanked by SNP markers rs758706 and rs1476878, with a maximum multipoint LOD score of 2.144 (θ = 0.00) between rs234 and rs887882, when penetrance was assumed to be 0.8 (Fig. 1B,C, Supplementary Table 1). The highest probability haplotype in the family was reconstructed using best haplotype from the Merlin and Cyrillic programs and some key recombinant events were identified (Fig. 1A). All affected offspring of the patient (II:2) carried the same pathogenic haplotype. However, recombination was observed between rs887882 and rs1476878 in the unaffected posterity (III:5). Compared with the pathogenic haplotype of patient (II:5), recombination was ascertained between rs758706 and rs10234074 in the unaffected descendent (III:6) of this patient. Together with the results, it was suggested that in this family, the pathogenic gene was located at a region between rs758706 and rs1476878, which was co-segregated with the phenotype in all examined patients with SCZ. Therefore, the haplotype analyses traced SCZ to chromosome 7q21.13-q22.3, which was found to span a 15.072-cM region (Fig. 1B).

### Exome sequencing and co-segregation analysis

**Figure 2:**
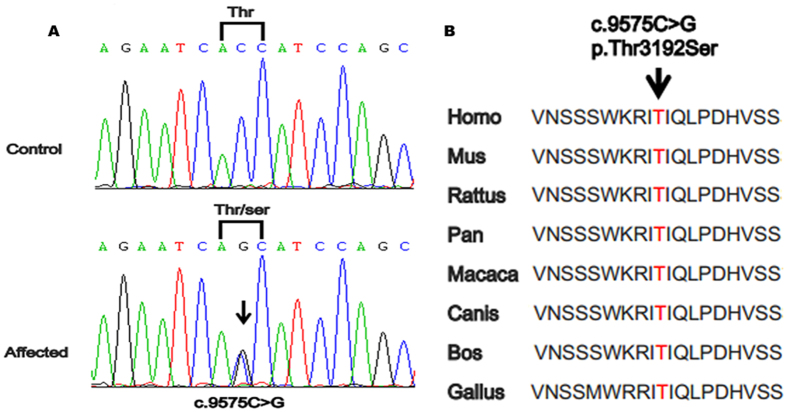
Heterozygous missense mutation of *RELN* in SCZ family and genomic organization of the human *RELN*. (**A**) Sequence analysis of exon 59 of *RELN* in a normal control and the affected proband of this family is shown. The DNA sequence chromatogram shows a heterozygous C > G nucleotide change (black arrow) in exon 59 of *RELN* (c.9575C > G), which leads to the replacement of threonine (ACC) with serine (AGC) at codon 3192 (p.Thr3192Ser). (**B**) Protein sequence alignment of *RELN* orthologs shows the region surrounding the mutated T3192S. Homo = *Homo sapiens*; Mus = *Mus musculus*; Rattus = *Rattus norvegicus*; Pan = *Pan troglodytes*; Macaca = *Macaca mulatta*; Canis = *Canis lupus*; Bos = *Bos taurus*; Gallus = Gallus domesticu.

Assuming that these three patients have the same genetic cause of disease reduced the number of variants to 6,106 common to all affected individuals. As the hereditary mode in the family is autosomal-dominant, the candidate variants were expected to be heterozygous in the three patients. To distinguish potentially pathogenic mutations from other variants, we focused only on functional variants (non-synonymous, nonsense, located in the canonical splice sites, or coding indels), anticipating that synonymous variants were far less likely to be pathogenic. On the basis of the genotypes of available public data-sets, additional polymorphisms were excluded, leaving 33 novel variants. Considering that most pathogenic variants either affected highly conserved sequences and/or were predicted to be deleterious, we used the ANNOVAR package, which contains 23 types of software to assess the remaining variants for a likely functional impact. As a result of the filtering steps above, the candidate pool was reduced to 19 variants (19 genes). To identify rare variants co-segregating with SCZ for the kindred, we have performed variant detection in all available members of the pedigree. The missense variant (c.9575C > G, p.Thr3192Ser) in *RELN* (chr7:103131145, RefSeq NM_005045/ NM_173054) was the only co-segregating mutation among these candidate variants tested by Sanger sequencing (Fig. 2A). The variant status of all tested individuals was depicted in Fig. 1A. Additionally, this variant was not found in the NHLBI Exome Sequencing Project (ESP6500), the 1000 Genomes Project, and the Single Nucleotide Polymorphism Database (dbSNP137), while the allele frequency of this variant was 8.24 × 10^−6^ in the ExAC database. Moreover, the missense substitute was also not detected in the 500 unaffected control individuals, indicating the rarity of the variation in the same geographic population. Thr3192 was found at a highly conserved position, indicating an important role for this residue. (Fig. 2B). Apart from these, the mutation we identified using exome sequencing is located in the same chromosomal region (7q21.13-q22.3) as that identified by linkage analysis, which cross-validated *RELN* as the potentially causative SCZ gene in this family (Fig. 1B).

## Discussion

In the Chinese family with autosomal-dominant SCZ, we identified a rare SCZ-related genetic variation (c.9575 C > G, p.Thr3192Ser) in *RELN* gene. It was detected using complementary genome-wide screening methods, validated by Sanger sequencing and rarely found in large sequencing databases of controls. To our knowledge, rare variations of *RELN* gene have not been detected in familial SCZ. Here, we conducted CNV study, linkage analysis and whole genome sequencing to confirm that the rare highly penetrant variation in *RELN* gene predisposed to SCZ in a Han Chinese pedigree. Similar to Timms *et al.* study^8^, we also didn’t discover any pathogenic CNV. Timms *et al.* provided one possible explanation that large rare CNVs confer risk for a more severe form of the disease (childhood onset or with associated comorbid intellectual disability), limiting reproductive fitness and subsequent familial transmission. Linkage analysis traced SCZ to chromosome 7q21.13-q22.3, which showed considerable overlap with the region reported by Ekelund *et al.* in Finnish SCZ patients^23^. Replication in independent studies demonstrates that the region 7q22 may contain a SCZ susceptibility gene, wherein, interestingly, *RELN* is located. Subsequently, the missense substitution of *RELN* was also identified via exome sequencing, further providing two separate experimental methods for localizing the gene of interest.

For sporadic SCZ, previous studies reported the contribution of rare variants and common variants to SCZ. Several exome sequencing studies in large SCZ cohorts already addressed the role of *RELN* rare variation in SCZ. For example, a rare missense variant (chr7:103194134 T > C, p.Tyr1981Cys) was detected in Bulgarian trio de novo exome sequencing SCZ sample^24^; some rare non-synonymous and disruptive mutations of *RELN* gene were found in Swedish SCZ patients^19^ (See Schizophrenia Exome Sequencing Genebook). On the other hand, common variants in *RELN* gene were also associated with SCZ. Firstly, SNP (rs2229860) has been described as a risk allele in relation to SCZ in a meta-analysis across three independent Caucasian case-control cohorts^25^. In addition, Shifman *et al.* reported a SNP (rs7341475) in *RELN*, which showed female-specific association with SCZ in Ashkenazi Jews^26^. This group also successfully replicated the association in a sample from the United Kingdom. Recently, rs7341475 was described as associated with the risk of paranoid SCZ^27^. Another common variant (rs362719) of *RELN* was also identified to be associated with SCZ in female Han Chinese^28^, but the results of association studies between *RELN* and SCZ were not exactly replicable in different populations. The reported association of rs7341475 in Ashkenazi Jews is not significant in Han Chinese^29^. These studies have illustrated that *RELN* is a convincing vulnerability factor for sporadic SCZ^25^,^26^,^27^,^28^,^29^,^30^,^31^.

*RELN*, located at 7q22 in humans^32^, is a strong susceptibility gene in diverse psychiatric diseases including SCZ, major depression, bipolar disorder, and autism^33^,^34^. *RELN* codes for reelin, which is a prominent component of the extracellular matrix and serves different functions during brain development and adulthood. During embryonic development, reelin is crucial for accurate cytoarchitecture of laminated structures^35^,^36^, demonstrated by reports of mutations in the *RELN* gene being associated with lissencephaly with cerebellar hypoplasia (LIS-CH)^37^. At postnatal and adult stages, reelin contributes to synapse formation^38^,^39^ and acts as a modulator of synaptic transmission and synaptic plasticity^40^,^41^,^42^. It is notable that in the postnatal brain, reelin is required for maintaining the mature subunit composition of N-methyl-D-aspartate receptors (NMDARs)^43^,^44^,^45^. Reelin binding to its receptors (very low-density lipoprotein receptor [VLDLR], apolipoprotein E receptor 2 [APOER2], and potentially a3b1 integrin) results in phosphorylation of NMDAR subunits, NR2A and NR2B. This process is mediated by the cytoplasmic adaptor protein Disabled-1 (DAB1) and Src-tyrosine kinase family/Fyn-kinase (SRC/FYN) activation^46^. Increasingly compelling reports suggest that synaptic dysfunction, particularly related to NMDAR signaling, is part of SCZ pathophysiology^10^,^11^,^12^,^13^,^14^,^15^,^16^,^17^,^18^,^19^,^20^,^21^,^22^,^23^,^24^. In addition, reelin is highly expressed in the normal human brain. However, a series of postmortem studies have demonstrated reductions in reelin protein levels at multiple brain sites (layers I and II of the prefrontal cortex, interstitial white matter neurons of the superior temporal cortex, hippocampus, and cerebellum) of subjects with SCZ when compared with controls^33^,^47^,^48^,^49^,^50^,^51^. Finally, animal models showing reelin deficit provide further evidence for symptoms of SCZ. Knockdown of reelin in the medial prefrontal cortex (mPFC) of Wistar rats during puberty or adulthood results in abnormal behavioral tasks with relevance to SCZ^52^. In a mouse model of prenatal restraint stress (PRS), offspring indicated a SCZ-like phenotype in behavior and displayed reduced expression of reelin in early life as well as adulthood, with increased DNA methyltransferase (DNMT) expression^53^. Teixeira *et al.* showed that a transgenic mouse model of reelin overexpression displayed resistance to developing psychiatric disorders such as SCZ, mood, and anxiety disorders, which indicated that reelin might have a protective role in these conditions^54^.

This study had certain limitations. To confirm statistical evidence for the observed variation, large case-control studies will be required to estimate its frequency and odds ratios (OR). Moreover, our study lacked functional evidences supporting the effects of the discovered variant. Furthermore, additional unrelated autosomal-dominant SCZ pedigrees were needed to evaluate whether other rare variants in *RELN* are responsible for familial SCZ.

Taken together, we identified a rare, missense mutation (c.9575 C > G, p.Thr3192Ser) within the RELN gene which might confer risk for SCZ in a Han Chinese pedigree. Our result provides genetic evidence that *RELN* can be implicated in the pathogenesis of SCZ.

## Subjects and methods

### Subjects

A total of three generations of a Chinese Han family with autosomal-dominant SCZ were enrolled in this study (Fig. 1A). The pedigree included 12 members, including 6 patients with SCZ. All affected individuals were diagnosed independently by at least two well-experienced psychiatrists according to the Diagnostic and Statistical Manual of Mental Disorders, Fourth Edition criteria (DSM-IV). Alternative conditions such as head injuries, substance-induced psychotic disorders, alcoholic psychosis, and other symptomatic psychoses have been excluded. Clinical data was collected by interviews and clinical questionnaires. A total of 500 unaffected individuals (mean age 30.0 ± 10.28 years) from the same geographic region as the schizophrenic patients, were recruited as normal controls. The study was approved by the Ethics Committee of Xiangya Hospital of the Central South University in China (equivalent to an Institutional Review Board) and carried out in accordance with the approved guidelines. A written informed consent was obtained from each subject. Genomic DNA was extracted from peripheral blood leukocytes using a standard salt precipitation protocol.

### CNV detection

To identify possible pathogenic CNVs, we examined genomic DNA samples from two affected individuals (II:5 and III:1) and one unaffected individual (II:3) in the family (Fig. 1A). The Human660W-Quad Chip (Illumina Inc., San Diego, USA) and the Illumina BeadScan genotyping system (Beadstation Scanner) were employed to obtain the signal intensities of single nucleotide polymorphism (SNP) probes following the manufacturer instructions. Genome Studio genotyping module software (Illumina) was used to analyze the genotypes (human genome build 37/Hg19) and evaluate experimental quality. The call rates of the samples were greater than 99.0%.

### Genome-wide genotyping for linkage analysis

To localize rare disease-related gene, we carried out whole-genome genotyping in the family using the Infinium HumanLinkage-12 Genotyping BeadChip (Illumina Inc., San Diego, USA). The BeadChip included 6090 SNP markers with an average gap of 441 kb and 0.58 cM across the genome. The genotype assignments were determined with Genome Studio genotyping module software (Illumina). Relative positions were derived from the human genome draft sequence and the Marshfield map (www.ncbi.nlm.nih.gov). Multipoint linkage analysis and reconstruction of the most likely haplotypes were performed using the linkage program, Merlin. The allele frequencies of markers, as well as the recombination fractions in males and females, were assumed to be equal. The disease was modeled as an autosomal-dominant trait with a disease allele frequency equal to 0.01. For calculating the logarithm of odds (LOD) scores, the disease penetrance was assumed to be 80%^55^ (Supplementary Table 1).

### Whole-exome sequencing and mutation sequencing

To identify the pathogenic mutation causing SCZ in this Chinese kindred, we performed exome sequencing analysis in three affected individuals (II:5, III:2, III:4) and one unaffected family member (III:5) (Fig. 1A). NimbleGen Sequence Capture Arrays were used to isolate the genomic coding regions to be sequenced. For enrichment, paired-end libraries were hybridized with the NimbleGen SeqCap EZ v2.0, which contains 2.1 million probes that capture a total of 44.1 Mb regions. Sequencing was subsequently performed using a HiSeq 2000 platform with 90-bp, paired-end reads. The mean coverage per targeted base was 70.10-fold. R20-fold coverage of bases was 89.2% for II:5, 92.1% for III:2, 90.1% for III:4, and 92.5% for III:5, respectively. The coverage of target region was 98.9%, 98.8%, 98.9%, and 99.1%, respectively. Clean data were obtained by removing adaptor and low quality reads, followed by aligning to the human genome reference assembly (USCS hg19) using Burrows-Wheeler Aligner (BWA). We performed data preprocessing involving removal of polymerase chain reaction (PCR) duplications using Picards, followed by indel realignment and base quality score recalibration by GATK. Haplotypecaller and variant quality score recalibration were adopted to identify the variants and recalculate their quality scores. This analysis identified a total of 29,964, 34,257, 35,754, and 34,746 variants, respectively, for II:5, III:2, III:4, and III:5. All variants were annotated with variants alleles frequency (VAF) in the NHLBI Exome Sequencing Project (ESP6500), the 1000 Genomes Project, and the Single Nucleotide Polymorphism Database (dbSNP137). Possible pathogenic variants were confirmed by Sanger sequencing using ABI 3730 DNA sequencer (PE Applied Biosystems), following the manufacturer protocol. Sequencing data was analyzed by DNA Star software (DNASTAR, Inc. Madison, Wisconsin, USA).

## Additional Information

**How to cite this article**: Zhou, Z. *et al.* Identification of *RELN* variation p.Thr3192Ser in a Chinese family with schizophrenia. *Sci. Rep.* **6**, 24327; doi: 10.1038/srep24327 (2016).

## Supplementary Material

Supplementary Dataset 1

## Figures and Tables

**Table 1:** Clinical features of six affected family members.

Patient	Gender	Age of onset (year old)	Hallucinations	Delusions	Disorganized speech	Abnormal psychomotor behavior	Negative symptoms (restricted emotional expression or avolition)	Depression	Mania	Loss of insight
II:2	Female	39	+	+	+	−	+	+	−	+
II:5	Female	32	+	+/−*	−	+	−	−	−	+
III:1	Male	31	−	−	+	+	+	−	−	+
III:2	Female	37	−	+	−	+	+	−	−	+
III:3	Male	35	−	+	−	+	+	−	−	+
III:4	Male	36	+	+	+	+	−	−	+	+

Note: **−**: not present, +: present, *uncertain.
