# Supplementary material for: Identification of RELN variation p.Thr3192Ser in a Chinese family with schizophrenia
Source: Sci Rep. 2016 Apr 13;6:24327. doi: 10.1038/srep24327 (PMC4829830; doi:10.1038/srep24327)
Supplement: Supplementary Dataset 1 [file srep24327-s1.docx]

**Supplementary data**

**Identification of *RELN* variation p.Thr3192Ser in a Chinese family with autosomal-dominant schizophrenia**

**Zhifan Zhou**^1,2^**, Zhengmao Hu**^3^**, Lu Zhang**^6,7^**, Zhaoting Hu**^1^**, Haihong Liu**^4^ **Zhening Liu**^5^**, Juan Du**^1,2^**, Jingping Zhao**^5^**,** **Lin Zhou**^1^**,** **Kun Xia**^3^**, BengshaTang**^1,2,3^**, Lu Shen**^1,2,3 *^

Table 1. Multi-point LOD scores were summarized.

| SNPs | Marshfield  cM | LOD scores by the disease penetrance（0.800） |
| --- | --- | --- |
| rs612774 | 98.16988 | -0.971 |
| rs1024516 | 99.01827 | 1.266 |
| rs4148738 | 99.39017 | 1.426 |
| rs1202169 | 99.404 | 1.428 |
| rs1688886 | 99.57595 | 1.434 |
| rs1637489 | 99.58009 | 1.434 |
| rs1468121 | 99.88965 | 1.445 |
| rs1023564 | 100.2987 | 1.446 |
| rs194518 | 101.019 | 1.444 |
| rs42611 | 101.0376 | 1.444 |
| rs758706 | 101.5913 | 1.443 |
| rs10234074 | 102.82 | 1.833 |
| rs722263 | 103.5034 | 1.888 |
| rs1859121 | 105.9523 | 2.022 |
| rs1053275 | 105.9705 | 2.023 |
| rs1917486 | 106.4638 | 2.046 |
| rs1229540 | 107.6216 | 2.094 |
| rs1047035 | 108.56 | 2.129 |
| rs219826 | 108.8836 | 2.141 |
| rs4727439 | 109.77 | 2.141 |
| rs1617640 | 110.019 | 2.141 |
| rs11178 | 110.4117 | 2.141 |
| rs727708 | 112.5311 | 2.142 |
| rs1010340 | 113.8075 | 2.142 |
| rs234 | 115.3992 | 2.144 |
| rs41261 | 115.5096 | 2.144 |
| rs176481 | 115.6852 | 2.144 |
| rs887882 | 115.8004 | 2.144 |
| rs1476878 | 116.6633 | 1.445 |
| rs257376 | 116.747 | 1.445 |
| rs2028030 | 117.7744 | 1.444 |
| rs441534 | 118.39 | 1.444 |
| rs10234165 | 118.6026 | 1.444 |
| rs1013920 | 119.5395 | 1.444 |
| rs719530 | 119.5934 | 1.444 |
| rs214459 | 120.0795 | 1.444 |
| rs214468 | 120.0831 | 1.444 |
| rs37742 | 120.0997 | 1.444 |
| rs1476517 | 120.287 | 1.444 |
| rs7817 | 120.5798 | 1.444 |
| rs2940339 | 121.075 | 1.444 |
| rs10279936 | 121.5891 | 1.444 |
| rs2040587 | 121.9733 | 1.444 |
| rs2056865 | 122.7709 | 1.444 |
| rs41736 | 123.2089 | 1.341 |
